# Modelling a Transition from Purebred Romney to Fully Shedding Wiltshire–Romney Crossbred

**DOI:** 10.3390/ani10112066

**Published:** 2020-11-07

**Authors:** Lydia Jane Farrell, Stephen Todd Morris, Paul R. Kenyon, Peter R. Tozer

**Affiliations:** School of Agriculture and Environment, Massey University, Private Bag 11 222, Palmerston North 4442, New Zealand; S.T.Morris@massey.ac.nz (S.T.M.); P.R.Kenyon@massey.ac.nz (P.R.K.); P.Tozer@massey.ac.nz (P.R.T.)

**Keywords:** New Zealand, profit, bio-economic, system-dynamics, wool, cashflow, flock dynamics, grading up, ewes, sheep

## Abstract

**Simple Summary:**

A flock of full-fleeced ewes could be bred to shed all their fleece through the repeated crossbreeding of ewes with shedding rams. This shedding flock would then not need to have their low value wool removed, thus reducing the associated expenses. This study used a simulation model to explore changes in the sheep numbers of different crosses, production, cashflow, and profit during the crossbreeding period. It took 12–15 years of crossbreeding to achieve a fully-shedding third or fourth cross flock. Economically, crossbreeding to a shedding flock compared favourably with farming a full-fleeced flock. More data on the performance of shedding sheep in New Zealand would improve the accuracy of model predictions.

**Abstract:**

Considering the current low prices for coarse wool (fibre diameter > 30 µm), a grading up transition to a shedding flock may eliminate wool harvesting costs and increase sheep farm profit. This transition could be achieved by breeding non-shedding ewes with Wiltshire rams. A bio-economic system-dynamics model of a pastoral sheep farming enterprise was used to simulate this grading up transition from 2580 Romney ewes to a similarly-sized flock of fully shedding third or fourth cross Wiltshire–Romney ewes. The total annual sheep feed demand was constrained within a ±5% range to minimise disruption to the on-farm beef cattle enterprise. Wool harvesting expenses were eliminated after seven years of transition, and with reduced feed demand for wool growth, the post-transition shedding flocks had more ewes producing more lambs and achieving greater annual profit compared with the base Romney flock. The net present values of transition were 7% higher than the maintenance of the base Romney flock with a farmgate wool price of $2.15/kg. Results suggest that coarse wool-producing farmers should consider a grading up transition to a shedding flock, and the collection of data on the production of Wiltshire–Romney sheep in New Zealand would improve the accuracy of model predictions.

## 1. Introduction

The majority of New Zealand sheep are dual-purpose breeds producing coarse wool (fibre diameter > 30 µm) and lambs for meat production, such as the dual-purpose Romney that makes up 52% of the national flock [1]. The majority (76%) of wool produced in New Zealand is exported, and coarse wool is sold at the world market price [1]. The nominal price for coarse wool has fluctuated between NZD 2.99 and $4.98/kg clean between 1990 and 2018 [1] (NZD = New Zealand dollars. All economic values reported for this analysis are in NZD). While the average fleece weight of New Zealand sheep has remained relatively stable over the period of 1990–2018, at 5.2 kg of greasy wool per head, the total industry wool production has declined during the same period alongside falling sheep numbers [1]. Sheep and beef cattle production usually take place on the same farms in New Zealand. Revenue from wool sales constituted on average 13% of gross cash income for New Zealand sheep and beef farms in 2007, which decreased to 7% in 2017, with the majority of income derived from sales of sheep and beef cattle for meat production [2,3]. Reductions in the economic importance of wool production for most New Zealand sheep farmers in recent decades has occurred alongside increasing shearing (the process of wool harvesting by shearing contractors) expenses [2]. Thus, some farmers now consider shearing an animal welfare necessity rather than a source of income.

Sheep breeds such as the Wiltshire and Dorper annually shed most or all of their fleece [4,5,6,7], reducing or eliminating shearing requirements and associated expenses. Partially or fully shedding sheep have been identified as potential options to improve animal welfare outcomes [8,9]. Shedding sheep typically initially lose wool from their breech [7,9,10] and have been reported to have lower incidences of animal health issues such as flystrike [6,11,12,13]. Heritability for fleece shedding has been reported to be relatively high (0.52) in New Zealand Wiltshire sheep [7,14]. This suggests that relatively quick progress in achieving a fully shedding flock could be made through breeding Wiltshire rams with Romney ewes. The selection of Wiltshire–Romney crossbred ewe lambs for greater shedding and their further backcrosses with Wiltshire rams could potentially achieve a fully shedding flock of predominantly Wiltshire shedding-type ewes within three-to-four generations through a grading up transition [4]. The scale of potential changes in production and profit during such a grading up transition are not currently known, nor are the length of time and number of generations necessary to achieve a whole shedding flock through crossbreeding non-shedding ewes with Wiltshire rams.

System dynamics modelling allows for the exploration and quantification of the dynamic impacts of feedbacks in a system, as previously demonstrated for New Zealand pastoral farm systems [15,16,17,18,19]. The authors of [18] used a bio-economic system-dynamics model of a New Zealand sheep farming enterprise to simulate a whole flock grading up transition from purebred Romney to second cross ¾Merino¼Romney, reducing wool fibre diameter to produce higher value wool. The objective of the current study was to use the same model to simulate a whole flock breed grading up transition from a purebred Romney to a third (⅞Wiltshire⅛Romney; ⅞W⅛R) or fourth (15/16Wiltshire1/16Romney or ‘straightbred’^2^) Wiltshire–Romney cross flock to achieve a fully shedding flock. System dynamics modelling was used in this analysis to capture flock dynamics with associated feed demand, production, cashflow, and profit implications before, during, and post-grading up transition.

## 2. Materials and Methods

The farm system under consideration was an East Coast North Island Hill Country sheep and beef farm in New Zealand. This farm system has both sheep and beef production enterprises, as well as potentially deer and non-lactating dairy cattle, where sheep account for 60% of the total feed consumed and the remainder is mostly consumed by beef cattle [2]. The farm was 530 ha with a self-replacing flock of 2066 mature Romney ewes lambing annually in spring and extensively grazing on pasture year-round. The sheep enterprise was the focus of this research, with the proportion of feed consumption accounted for by sheep used to estimate operating expenses and farm area applicable to the sheep enterprise. The Romney flock was based on average values from industry survey data for East Coast North Island Hill Country farms in the 2017/18 production year [2].

The bio-economic system-dynamics model was constructed using STELLA version 1.9.3 [20] with separate component modules for each ewe flock’s dynamics, sheep feed demand on a fortnightly basis, and wool production. Feed supply and economics were estimated in component modules for the entire farm. More detail on model workings were reported in [16,17,18,19]. This study extended the previous model to include the option of crossbreeding to a third (⅞W⅛R) and/or fourth (straightbred) cross ewe flock. In order to capture changes in wool production and shearing expenses when integrating shedding genes into the flock, the wool production and economics modules were also updated. The total sheep feed demand as a proportion of the total farm feed supply had a base level of 60% [2], with sheep numbers and production constrained during transition to maintain sheep feed demand between approximately 55% and 65% of the total farm feed. This constraint on sheep feed demand was assumed to maintain a relatively constant proportion of feed consumed by the on-farm beef herd.

### 2.1. Flock Dynamics

**Figure 1:**
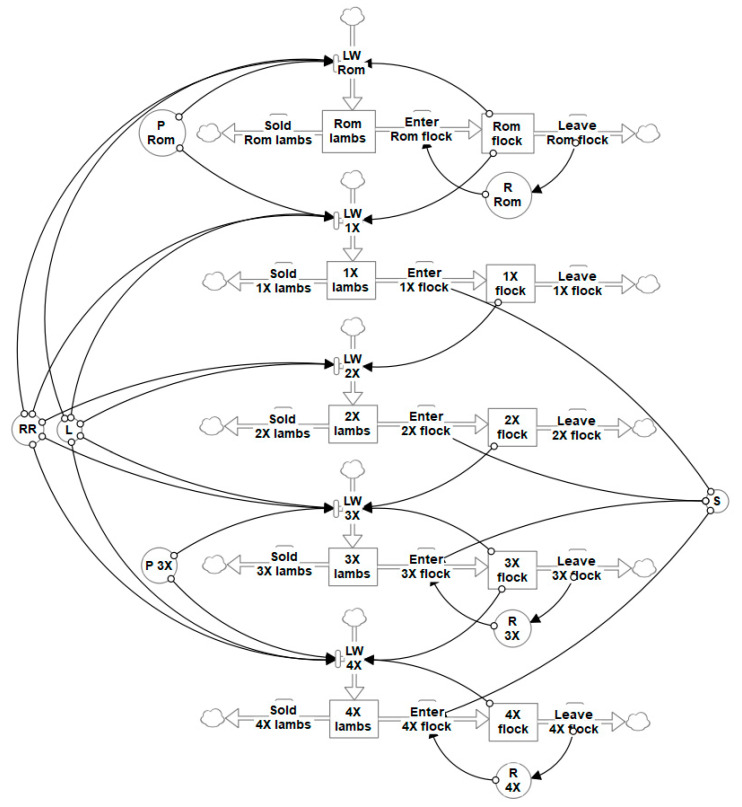
Simplified diagram of the flock dynamics for a flock grading up transition from purebred Romney (Rom) to either a third (3X or ⅞W⅛R) or fourth (4X or straightbred) generation crossbred. Lambs weaned (LW) for each cross are a product of *P* (proportion of ewes bred with a Wiltshire sire), L (lambs weaned per ewe presented for breeding), flock (number of ewes in the flock), and RRi (relative reproductive performance of ewes for each age class in the flock)—where *P* = 1 produces crossbred lambs closer to a Wiltshire breed than their dams to further the grading up transition. Rom, 3X, and 4X flocks can be self-replacing when *P* = 0 by calculating replacement ewe lamb requirements (R) based on numbers of ewes leaving to flock due to culling and death. First (1X or ½Wiltshire½Romney (½W½R)) and second (2X or ¾W¼R) cross flocks can only produce lambs of further crosses (and 3X flock will produce only 4X lambs if the 4X flock is the desired final cross). The selection intensity (S) of crossbred ewe lambs determines the proportion that enter the crossbred flocks, with all remaining lambs sold ^1^. ^1^ The symbols in the diagram are as follows: rectangles represents stocks (or groups) of sheep of a specific age and breed; white arrows are flows representing the movement of sheep between groups or, where there is a cloud, entering or leaving the flock; circles are convertors with functions affecting flows; and black arrows are connectors that join stocks, flows, and convertors.

A simplified diagram of the flock dynamics component module is shown in Figure 1, demonstrating how the initially self-replacing Romney flock could be modelled to produce crossbred lambs to initiate the grading up transition. It is not currently known if a fully shedding flock would be achieved in Wiltshire-based crosses of either ⅞W⅛R or straightbred. This study modelled to a grading up transition end point of either ⅞W⅛R or straightbred as separate scenarios. The flocks of these final composites were then modelled as self-replacing once they reached an equivalent feed demand to the base Romney flock.

#### 2.1.1. Flock Dynamics of Self-Replacing Romney Flock

Only the sheep operations of the farm were analysed in this model as with previous modelling by [16,17,18,19]; producing coarse wool, prime lambs and cull ewes sold direct to slaughter, and store lambs sold to be finished on another farm [2]. Ewes in each age (*_i_*) class (*Y_i_*) each year were the sum of ewes in the previous age class (*Y*_*i*−1_) minus ewes leaving the flock due to deaths (*D*_*i*−1_) and culling (*C*_*i*−1_) (Equation (1)). The ewe flock was therefore the sum of ewes in six age classes (Equation (2)). When a self-replacing flock was modelled, it was held at a constant size with the flock replacement requirements (*R*) calculated as the sum of all ewes leaving the flock due to death and culling (Equation (3)). Death rates of 5.2% for *Y*_2 *to* 6_ ewes and 2% for *Y*_1_ ewes were assumed [2]—this included missing ewes, with a flock replacement rate of 25% that is typical of New Zealand sheep breeding flocks [21]. All ewes in *Y*_6_ were culled after their lambs were weaned.

(1) $$Y_{i}=Y_{i-1}-D_{i-1}-C_{i-1}$$

(2) $$\mathrm{And}\;Y_{1\;to\;6}=\sum_{i=1}^{6}Y_{i}$$

(3) $$\mathrm{And}\;R=\sum_{i=1}^{6}[D_{i}+C_{i}]$$

The numbers of lambs weaned (LW) were estimated from Equation (4) as a function of the ewes of each age class presented for breeding (*Y_i_*), *L* (the average flock lambing rate as lambs weaned per ewe presented for breeding; 132%) [2], *P* (proportion of ewes bred with a Wiltshire sire, i.e., when modelling the self-replacing Romney flock *P* = 0 to produce only purebred Romney lambs), and the relative reproductive performance for each ewe age class (*RR_i_* peaking in *Y*_5_, as detailed in [16]). The number of lambs born as singles and twins depended on whole flock reproductive performance [22]. Sheep farmers in New Zealand choose whether to first breed ewes to lamb at 14 months or two years of age; in this study, it was assumed that ewes had their maiden lambing at two years of age (*Y*_2_), so *RR*_1_ = 0.

(4) $$LW=\overset{6}{\underset{i=1}{\sum }}\left[Y_{i}\times L\times RR_{i}\times \left(1-P\right)\right]+\overset{6}{\underset{i=1}{\sum }}\left[Y_{i}\times L\times RR_{i}\times P\right]$$

#### 2.1.2. Flock Dynamics During Transition

To initiate the grading up transition, the parameter *P* in Equation (4) was set to *P* = 1 for the Romney flock to breed only ½Wiltshire½Romney (½W½R) lambs (Figure 1). The ½W½R and ¾Wiltshire¼Romney (¾W¼R) flocks could only be bred with Wiltshire rams to continue the grading up transition to a ⅞W⅛R flock. If the desired final cross was ⅞W⅛R, then *P* was set to *P* = 0 for the ⅞W⅛R flock. If the desired final cross was a fourth cross straightbred flock, then *P* = 1 for the ⅞W⅛R flock. The label of ‘desired final cross’ refers to both of the separate grading up transition scenarios to either a ⅞W⅛R or straightbred flock. The straightbred flock was considered to be a stable Wiltshire breed, so the grading up transition could not continue beyond the straightbred cross. Equation (4) was used to estimate lambs weaned (*LW*) of each cross, with *L* and *RR_i_* consistent between crosses. 

Thus, differences in numbers of lambs weaned of each cross were due to differences in *P* and the size and age structure of ewe flocks (*Y*_1 *to* 6_). The size and age structure of crossbred flocks during transition were determined primarily by ewes entering (such as ewe lambs entering the *Y*_1_ age class according to Equation (5) and then aging according to Equation (1)) and leaving the flock. Numbers of crossbred ewe lambs entering *Y*_1_ during transition were determined by the selection intensity (*S*) of 50% applied to all weaned crossbred ewe lambs after shedding scoring in January (Equation (5)). Lambs were approximately five months old in January, which has been identified as the best time to assess fleece shedding in New Zealand lambs [7]. The selection event occurred for crossbred ewe lambs each year, and ewe lambs with the highest shedding scores remained on-farm to enter crossbred ewe flocks. Crossbred ewe lambs not selected, and ram lambs were sold prior to winter.

(5) $$Y_{1}=LW\times 0.5\times \left(1-S\right)$$

##### Ewe Culling during Transition

Ewes left the crossbred flocks due to culling and deaths, with a death rate of 5.2% for *Y*_2–6_ ewes and 2% for *Y*_1_ ewes, consistent with the self-replacing Romney flock. The numbers of ewes in each age class and flock were estimated according to Equations (1), (2), and (5) for all crosses. Cull rates differed between the flocks of various crosses during transition in order to limit the changes in the total annual sheep feed demand and to hasten the time taken to replace the base Romney flock with an approximately equivalent flock of the desired final cross (Table 1). Cull rates were 4% for *Y*_2–3_ ewes of all crosses during the grading up transition, assuming that only barren ewes in these age classes were culled [23]; this assumed cull rates were minimised when aiming to increase ewe numbers for the desired final cross. For the self-replacing Romney flock pre-transition and the flock of the desired final cross (either ⅞W⅛R or straightbred), there were six age classes of ewes, with all *Y*_6_ ewes culled after weaning. When modelling a grading up transition, flocks not of the desired final cross (not either ⅞W⅛R or straightbred) only had five age classes of ewes, with all *Y*_5_ ewes culled after weaning and no ewes in *Y*_6_. Additionally, to maintain a consistent annual sheep feed demand, the cull rate was varied for ewes in *Y*_4_ for different crosses (Table 1), and at specific time points, all remaining ewes not of the desired final cross were culled (timings shown in results). Once the ewe flock of desired final cross (⅞W⅛R or straightbred) achieved the same feed demand as the base Romney flock, it was modelled as a self-replacing flock (Figure 1) with six age classes and a replacement rate of approximately 25% calculated from Equation (3).

### 2.2. Wool Production

Wool produced by the base Romney flock was assumed to have an average fibre diameter of 36 µm and an average mature ewe greasy fleece weight of 5.2 kg (*W*—where *W* for crossbred sheep changed with their shedding score) [1]. All Romney lambs on-farm in January were assumed to be shorn along with the ewe flock, with the total flock wool production estimated according to mature fleece weight (*W* in kg), an adjustment parameter for the fleece weight in each age class (*w_i_*; *w*_0.5_ = 0.50, *w*_1_ = 0.95, *w*_2_ = 1.01, *w*_3_ = 1.08, *w*_4_ = 1.05, *w*_5_ = 1.01, and *w*_6_ = 0.97) [24,25,26] and the numbers of sheep in each age class (*Y_i_*) in Equation (6). The wool production of the Wiltshire–Romney crossbred flocks was also estimated using Equation (6), with *W* altered according to shedding score.

(6) $$WP=\sum_{i=0.5}^{6}Y_{i}\times \left(W\times w_{i}\right)$$

#### Wool Shedding

The proportion of fleece shed by crossbred sheep in this study was expressed using the shedding score system utilised in New Zealand by [7,14], with scoring occurring in January at five months of age. A score of zero was given to sheep with no evidence of shedding, and a score of five denoted a sheep fully shedding its fleece, with the progression of shedding starting with the belly and breech (score of one), and eventually with the wool left along the spine (score of four) before all wool was shed (score of five). The current study assumed a sheep with a score of 2.5 to have shed approximately half of its fleece, so a score of four indicated that 80% of the fleece had been shed.

Experimental data indicate that crossbred offspring with various levels of shedding genes would have shedding scores similar to their parental average [4,10]. The same studies included data on the variation of shedding in offspring, with variance estimates of 38%, 8%, and 2% for first, second, and fourth crosses, respectively, from which a variance of 5% could be deduced for a third cross. These variance estimates, a parental average, and normal distribution were assumed for the shedding scores of ewe lambs of each cross in order to estimate a new average shedding score after selection each January. This effect of selection for shedding was estimated with the same methods as those used in [18] to simulate selection for a reduced wool fibre diameter.

**Figure 2:**
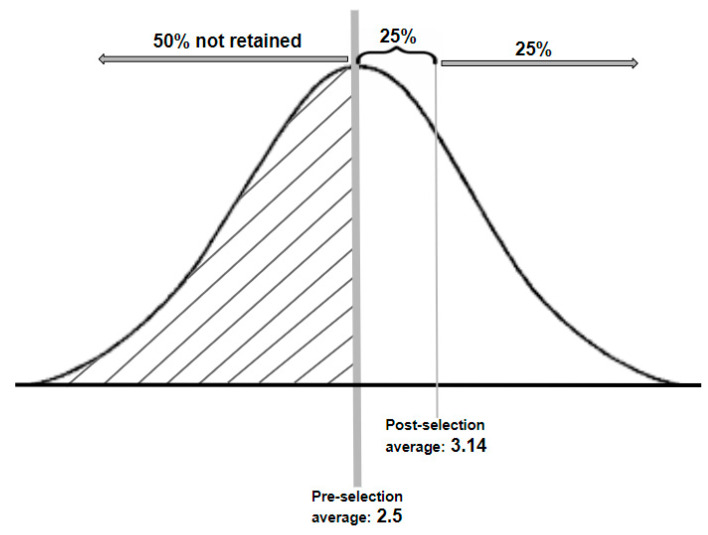
Simplified example of the effect of a selection intensity of 50% on the average shedding score in ½W½R ewe lambs from Romney dam with a score of 0 and Wiltshire sire with a score of 5.

Figure 2 is a simplified illustration of an example of selection for greater shedding in first cross ½W½R ewe lambs. The pre-selection the lambs had an average shedding score of 2.5 (from a Wiltshire sire with a score of five and Romney dam with a score of zero), with a standard deviation of 0.95 (38% variance). When a selection intensity of 50% was applied, lambs with a shedding score lower than the average were not retained (see the shaded section to the left of the average). The post-selection average would therefore be the mid-point of the remaining ewe lambs (50% of weaned ewe lambs); in this example, 25% of the area under the curve was above the post-selection average, which resulted in a new average score of 3.14. Studies of shedding sheep reporting both the extent of shedding and greasy fleece weight were used to estimate the amount of wool remaining on sheep for harvesting (W) with various shedding scores [4,14]. These data, as well as an assumed greasy fleece of 5.2 kg for non-shedding Romney ewes [1], were used to derive Equation (7), where C is the shedding score that was used to estimate harvestable fleece weight of crossbred ewes.

(7) W=−1.07×C+5.10

This study assumed that crossbred ewe lamb shedding scores at five months of age in January would be consistent with their future shedding scores. Therefore, we assumed that the selection for higher shedding scores in lambs in January would result in a permanent increase in shedding scores for crossbred ewes, as is shown in Table 4. However, studies of shedding sheep have consistently indicated that shedding increases with age so shedding in lambs is not a perfect predictor of shedding in adult ewes [7,12,27]. The lower wool shedding of lambs was accounted for in the current analysis because wool was not harvested or sold from crossbred lambs. There may be a risk of not retaining crossbred ewe lambs who would have displayed greater shedding as an adult, but this was not included in the analysis. However, the ability to select for shedding in crossbred ewe lambs at five months of age is important because lambs that are not selected can be subsequently sold prior to winter along with ram lambs.

### 2.3. Feed Demand

Sheep feed demand was estimated in the megajoules of metabolizable energy required for each group (i.e., cross, age class, and sale policy of lambs) of sheep according to their production and equations from [28,29]. Feed demand for maintenance, wool growth, gestation, lactation, and liveweight gain were estimated for all sheep and detail is provided in the Supplementary Materials. Without any available data on which to base an assumption around the weight of shed wool, its production was not included in this analysis (i.e., the feed demand for the growth of shed wool was not included). There were insufficient data to support assumptions of differences in the production and feed demand of the different breeds—lambing rate (132%), mature ewe liveweight (65 kg; [30]), lambing date (1 September; [3]), lamb loss rates (16% between pregnancy scanning and weaning; [23]), birth weights (5.5 and 4.5 kg for single- and multiple-born lambs, respectively; [31,32]), weaning weights (30 and 28 kg for single- and multiple-born lambs, respectively; [32,33]), and weaning age (12 weeks; [32,34]), which informed the feed demand equations, were assumed not to differ between the sheep of different crosses.

Lamb post-weaning growth rates and sale policies were also consistent across crosses. Lamb growth rates were assumed to average 100 g/day for all crosses [35,36]. The first group of prime (sold direct to slaughter) lambs were sold in early February with a carcass weight of 18 kg [2] and a dressing out rate of 41% [37,38] and a second group of prime lambs with the same characteristics were sold two weeks later. A group of store lambs (sold for another farmer to finish) was also sold in mid-February with a liveweight of 30 kg, lighter than would typically be sold prime [39]. For flocks of all crosses, 45% of the total lambs sold were sold store [2], and the numbers of prime lambs sold in each group were equal (i.e., 27.5% of the total lambs sold in each prime lamb group). Feed demand for the liveweight gain of all sheep, including lambs destined for sale, was estimated according to the equation from [29]. Feed demand for sold lambs’ maintenance was calculated based on the equation in the Supplementary Materials from [28] and their average liveweight during their time on-farm between weaning and sale.

### 2.4. Economics

The annual sheep enterprise cash operating surplus (COS) was used as an indicator of changes in profit and was estimated from gross sheep enterprise cash income (sheep and wool sales) and sheep-related operating expenses. The sheep enterprise COS was expressed on a per ha basis, with the COS therefore divided across the sheep proportion of the farm and sheep accounting for 60% of the total farm area of 530 ha pre-transition [2].

#### 2.4.1. Income

Prices for sold sheep, cull ewes, and sold lambs were from 2017/18, as shown in Table 2, and were consistent between crosses. A 2017/18 farmgate wool price of $2.15/kg greasy [2] was used to estimate wool income, with all wool assumed to be coarse.

#### 2.4.2. Expenses

Animal health expenses were estimated as $6.00 per stock unit [2], where wintered sheep stock units included ewes, rams, and replacement lambs. A stock unit is the equivalent of one 55 kg ewe weaning one 28 kg lamb, equal to an annual feed consumption of 550 kg DM (dry matter) [40]. General operating expenses were also estimated on a per stock unit basis at $47.80 per stock unit [2] and excluded expenses related to animal health, shearing, rates, rent, interest, and depreciation. It was assumed that breeding expenses were included in general operating expenses and that prices for Wiltshire rams were similar to Romney rams used within the base flock.

In order to capture potential expenditure reductions with reduced shearing requirements, expenses for shearing differed according to shedding score (Table 3). Shearing requirements were twice yearly shearing and crutching (crutching is the removal of wool from the belly and breech) for sheep with a score of zero (a full fleece such as the base Romney sheep), and no crutching was required for sheep with a score of one (assuming sheep first shed wool from the belly and breech). Shearing requirements were reduced with increased shedding score until sheep with a score of four were achieved, where it was assumed most wool was shed and none required shearing. It was assumed that Romney lambs on-farm in January were shorn at an expense of $3.71 per head [41], while Wiltshire–Romney crossbred lambs shed some of their wool and were not shorn.

#### 2.4.3. Net Present Value Analysis

In order to compare the scenarios as options for investment, net present value (NPV) analyses were undertaken using Equation (8) from [42]. NPVs capture the time value of cashflow during the grading up transition period, accounting for the timing of peak and the nadir total farm COS (Equation (9)), which differed between the modelled scenarios. The NPV analyses were conducted for each transition scenario and the maintenance of the base self-replacing Romney flock. The total farm COS was estimated each year using Equation (9). The sheep enterprise COS (*COS_Sheep_*) on a per ha basis was calculated based on changes in sheep numbers and production. A stable beef enterprise COS (*COS_Beef_*) of $81.24/cattle stock unit was assumed [2]. The grading up transition period was up to 15 years and included the total time taken for the flock of desired final cross ewes to reach a size with a similar feed demand to the base Romney flock. Changes in the numbers of ewes in each age class of the desired final cross flock occurred up until approximately 25 years from the beginning of the grading up transition, affecting flock productivity and COS. Therefore, NPV analyses were also conducted for 25-year periods.

(8) $$NPV=\overset{15\;\mathrm{or}\;25}{\underset{t=1}{\sum }}\frac{Total\;COS_{t}}{{\left(1+r\right)}^{t}}$$

(9) $$\mathrm{And}\;Total\;COS=[{Feed}_{Sheep\times }{COS}_{Sheep}+(1-{Feed}_{Sheep})\times {COS}_{Beef}]\times Area$$

where *Feed_Sheep_* is the proportion (0 ≤ *Feed_Sheep_* ≤ 1) of the total farm feed consumed by sheep (60% for the base Romney flock), and *Area* is the farm total effective area of 530 ha [2]. *Feed_Sheep_* varied during the grading up transition between approximately 55% and 65% according to changes in the total annual sheep feed demand. Additionally, *t* = each year during the time period analysed, either the grading up transition period only or 25 years from transition start. Discount rates (*r*) of 10% to reflect long-term New Zealand business lending interest rates [43] and 6% to reflect current lower interest rates, i.e., 2017/18 rates [44], were used. Economic values in this analysis were all in real 2017/18 terms, and the discount rates represented the real opportunity expenses for farmers investing in the grading up transition scenarios investigated.

#### 2.4.4. Wool Price Sensitivity Analysis

The main part of this analysis used a coarse wool price of $2.15/kg greasy, the farmgate price received by New Zealand East Coast North Island Hill Country farmers in the 2017/18 production year under study [2]. Separate NPV analyses were conducted with adjusted wool prices to explore how the potential economic benefits of a grading up transition change with wool returns reflecting recent and possible future prices, where all other parameters were unchanged. Wool prices were adjusted higher to $3.15 and $4.15/kg to explore how increased wool prices impacted the benefit of the grading up transition scenarios modelled. In August 2020, prices received at auction for coarse wool were as low as $1.70/kg clean and lower for wool from a second shear [45]. Therefore, NPV analyses with a farmgate wool price of $1.15/kg greasy were also conducted.

#### 2.4.5. Operating Profit

In this analysis, the COS was used to estimate changes in profit and cashflow for the various modelled scenarios. Estimates of the COS did not capture changes in farm capital or taxable profit during the grading up transition, specifically changes in the capital value of livestock with changing sheep numbers. Annual farm operating profit (OP) was estimated using Equation (10) from [46].

(10) $$OP=Total\;COS-M-Dep+\Delta V_{Sheep}+\Delta V_{Beef}$$

where the Total annual COS was estimated using Equation (8), *M* was managerial salary and drawings summing annually to $125,082, and *Dep* is an annual depreciation expense of $21,444 [2]. The value of sheep capital *V_Sheep_* was estimated using 2018 national average market values of $123 for replacement ewe lambs, $179 per one-year-old ewe, $160 per ewe aged two-to-six years, and $289 per ram [47]. A base *V_Beef_* value of $441,438 was adjusted in accordance with changes in the proportion of farm feed consumed by sheep. Changes in *V_Sheep_* and *V_Beef_* for annual OP were derived from the capital value of the current year (closing value) minus the capital value of the previous year (opening value). The Δ*V_Sheep_* = 0 and Δ*V_Beef_* = 0 for the base self-replacing Romney flock when it was assumed stock numbers were steady.

## 3. Results and Discussion

The grading up transition was completed when the flock of desired final cross ewes (either ⅞W⅛R or straightbred) had a similar feed demand to the base Romney flock, 12 and 15 years of transition for a desired final cross of ⅞W⅛R and straightbred, respectively. The numbers of ewes in each age class continued to fluctuate until around 25 years from transition start, which affected sheep feed demand, production, and COS; after 25 years, ewe numbers fluctuated annually by approximately ±5%. Therefore, the model was run for 25 years for each scenario, and results are reported for this period.

### 3.1. Shedding Score

**Figure 3:**
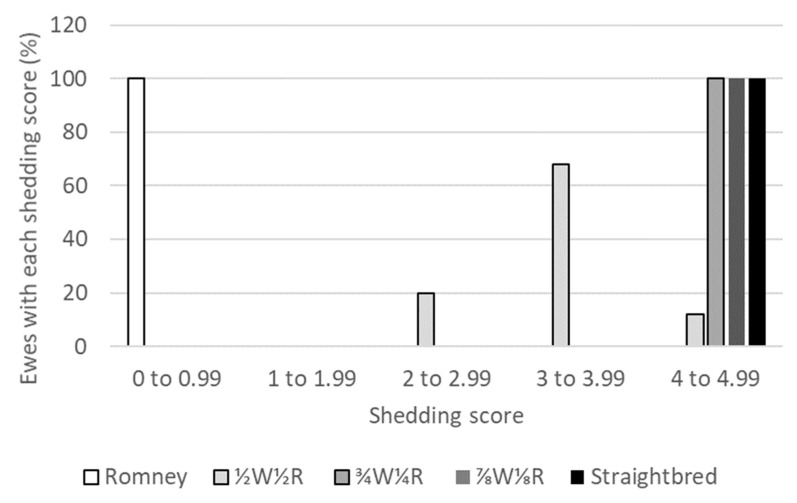
Proportion of ewes of each Wiltshire–Romney cross (e.g., ½W½R was ½Wiltshire½Romney and straightbred was assumed to be a stable Wiltshire breed) with each shedding score.

With a pre-selection average ewe lamb shedding score of 2.5 and an applied selection intensity of 50%, 20% of first cross ½W½R ewes in the flock had a score between 2.5 and 2.99, 68% had a score between 3.0 and 3.99, and 12% had a score greater than 4.0 (Figure 3). A ewe flock not requiring any shearing (shedding score greater than four) was achieved with a ¾W¼R second cross in this analysis. Further grading up was modelled as this would reduce the risk of expression of non-shedding genes in ewes of the desired final cross (either ⅞W⅛R or straightbred) post-transition.

The first cross ½W½R offspring had an average pre-selection shedding score similar to their parental average, and the shedding score increased to 3.14 with an applied selection intensity of 50% (Table 4). The shedding score variance of 38% assumed for ½W½R offspring was relatively larger than the variances assumed for further crosses (¾W¼R, ⅞W⅛R, and straightbred) which were less than 10%. This allowed for a greater change in shedding score in the ½W½R offspring with the same selection intensity applied (50% of crossbred ewe lambs retained). The shedding scores and variances used in this study were derived from relatively few data from studies of Wiltshire crosses farmed in the United Kingdom [4,10]. The collection of further data on the shedding scores and associated variances of Wiltshire crossbred offspring in New Zealand would improve the ability of the model to predict changes in wool shedding for a Wiltshire grading up transition.

Some New Zealand Wiltshire sheep are descendants of crossbreeding of fully shedding Wiltshire Horn and non-shedding Poll Dorset breed sheep; therefore, some New Zealand Wiltshire sheep may not fully shed their fleece [48]. The current analysis assumed that Wiltshire rams had shedding scores of 5.0. In a potential future analysis, if Wiltshire rams used in a grading up transition have a shedding score of 4.0 (rather than 5.0, as was assumed in the current analysis), an average shedding score of 3.97 would be achieved by ewes in the subsequently derived straightbred flock (fourth cross) with an applied crossbred ewe lamb selection intensity of 50% (all straightbred ewe lambs with scores below 3.92 not selected to enter the ewe flock). With an average score of only 3.97, it is likely that the majority of ewes would still require the removal of some wool from their back with shearing expenses of $2.00 per head (Table 3), which would impact the sheep COS for the Wiltshire–Romney crossbred flocks. Alternatively, a fully shedding flock could be achieved after a longer grading up transition, potentially with a higher selection intensity. However, grading up transition scenarios using Wiltshire rams with shedding scores of only 4.0 were not modelled in this analysis.

A normal distribution was assumed in this analysis for the shedding scores of crossbred ewe lambs prior to selection for shedding. This normal distribution was used to predict the change in average shedding score with an applied selection intensity of 50% (Figure 3 shows the distribution of shedding scores pre- and post-selection). Published data from Wiltshire–Blackface first and second cross progeny from [4] informed the assumptions of the average shedding scores for Wiltshire–Romney cross ewe lambs in the current study. The distribution of shedding scores of Wiltshire–Blackface cross sheep [4] were approximately symmetrical for second cross ewe lambs, slightly negatively skewed for adult first cross ewes, and positively skewed for second cross adult ewes. Data from Romane [49] and the crossbreeding of Katahdin, Romanov, and Dorper sheep [50,51] have demonstrated non-normally distributed shedding scores in lambs, though these animals are different in crossbreeding and parental breed characteristics to those modelled in the current study. These breeds are either not present on New Zealand commercial sheep farms or would not be utilised for the modelled grading up transition due to health issues. Therefore, data from [49,50,51] were not used for the assumptions in the current modelling. If shedding scores of New Zealand Wiltshire–Romney cross ewe lambs were found to not be normally distributed, the post-selection average shedding score would possibly differ from those predicted in this study, which could affect the time taken to reduce shearing expenses. More data on Wiltshire–Romney cross sheep in New Zealand is needed and would improve the ability of the model to predict changes in shedding score for the grading up transition under study.

### 3.2. Flock Dynamics

**Figure 4:**
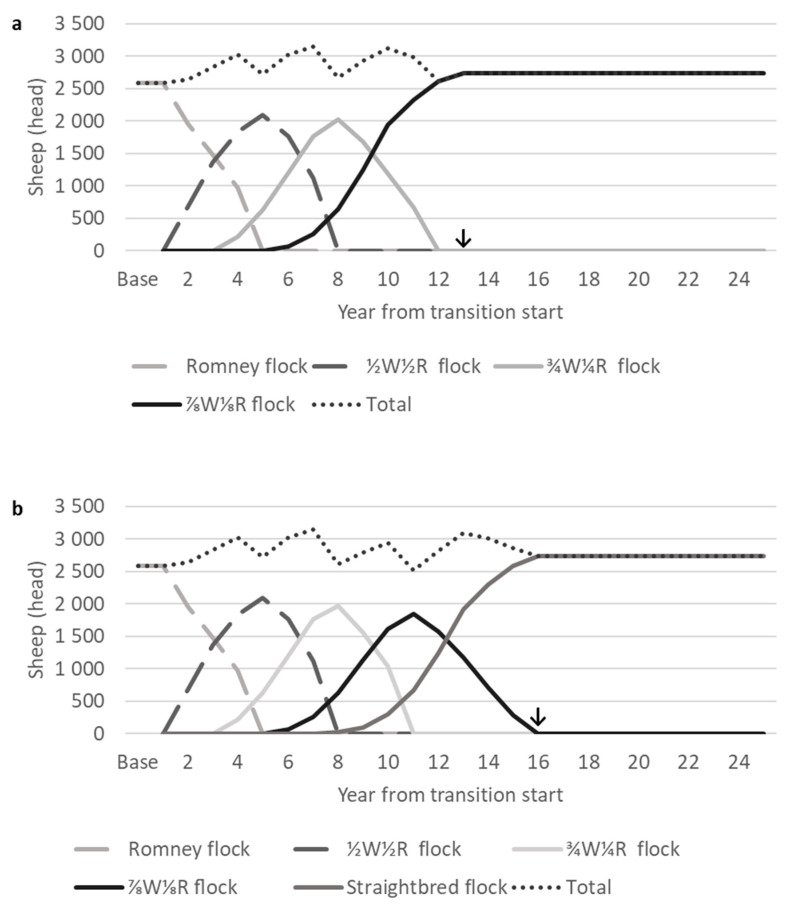
Numbers of ewes of each cross and total ewe numbers during a grading up transition from base Romney to either (**a**) ⅞Wiltshire⅛Romney or (**b**) straightbred. ↓ Where the grading up transition period has finished and the desired final cross flock (either ⅞W⅛R or straightbred) has reached an equivalent feed demand to the base Romney flock.

A shedding flock with a similar annual total sheep feed demand to the base Romney flock was achieved after 12 or 15 years of crossbreeding when transitioning to either a ⅞W⅛R or straightbred flock, respectively (Figure 4). The previous modelling of a whole flock transition to a second cross ¾Merino¼Romney by [18] predicted a transition period of 7–10 years. Therefore, the current longer period of transition to third and fourth crosses with constrained sheep feed demand was expected.

#### Ewe Age

The base Romney flock had an average age of 3.45 years; without replacement ewe lambs entering the flock during the grading up transition (as only crossbred lambs were produced), the average age of the Romney flock increased to 4.46 years before all remaining Romney ewes were culled (Table 5). During the transition to the desired final cross of ⅞W⅛R, the ½W½R and ¾W¼R flocks each reached an average age of over four years before all remaining ewes of their crosses were culled. During the transition to a straightbred cross, all remaining ¾W¼R ewes were culled earlier from the transition start, and so the ¾W¼R flock only reached an average age of 3.77 years before all remaining ¾W¼R ewes were culled. When transitioning to the straightbred flock, ⅞W⅛R ewes remained on-farm until up to year 15 from the transition start and therefore reached an average age of 4.99 years before all remaining ⅞W⅛R ewes were culled.

The flocks of the desired final cross each had average ages of more than 3.5 years when transition finished either 13 or 16 years from the start of the transition (Table 5). At 25 years from transition start, the average age of each self-replacing flock of desired final cross ewes had decreased while remaining close to the self-replacing base Romney flock pre-transition average age of 3.45 years. The reason for this lower age was that numbers of ewe lambs entering the flocks had decreased which decreased numbers of ewes in older age classes once numbers of ewes in each age class had stabilised. The difference in numbers of ewe lambs entering the flocks of desired final cross ewes was due to the different criteria for their entry. At the end of transition period, 50% of crossbred ewe lambs were still retained (873 and 859 ewe lambs for transition to ⅞W⅛R and straightbred flocks, respectively). Meanwhile, at 25 years from transition start, the numbers of ewe lambs entering the flocks were lower, only reaching those required to achieve a replacement rate of approximately 25% (740 and 688 ewe lambs for post-transition self-replacing ⅞W⅛R and straightbred flocks, respectively).

### 3.3. Feed Demand

**Figure 5:**
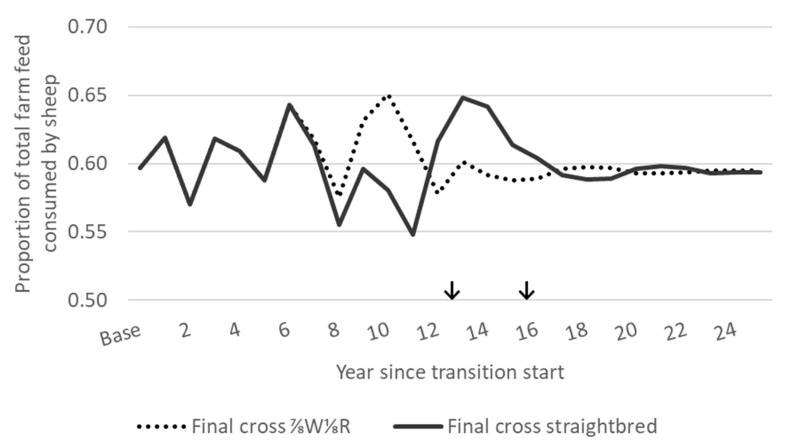
Proportion of total farm feed supply consumed by sheep, constrained between 0.55 and 0.65 of total farm feed supply, for a grading up transition from base Romney to a desired final cross of either ⅞W⅛R or straightbred. ↓ Where the grading up transition period has finished and the desired final cross flock (⅞W⅛R or straightbred) has reached an equivalent feed demand to the base Romney flock.

The total annual sheep feed demand was similar in the post-transition self-replacing ⅞W⅛R and straightbred flocks to that of the base Romney flock at approximately 60% of the total farm feed (Figure 5). This was achieved higher greater ewe numbers in the post-transition flocks (2736–2740 ewes) compared with the base Romney flock (2580; Figure 4). The larger flock size of ⅞W⅛R and straightbred ewes was due to their lower feed demand for wool growth. The daily feed demand for wool growth of ewes in the Romney flock of 2580 ewes averaged 1.07 MJ ME, resulting in an annual feed demand of just over 1 million MJ ME for wool growth. With a daily maintenance feed demand of approximately 10.5 MJ ME for 65 kg ewes on hill country [28,29], the additional 156–160 ewes in the ⅞W⅛R and straightbred post-transition flocks would have a total annual feed demand for maintenance of approximately 600,000 MJ ME. The remaining difference of 400,000 MJ ME was accounted for by additional feed demand for the gestation, lactation, and growth of the lambs from the 156 to 160 additional ewes. For example, the additional lambs would have a total annual feed demand for gestation and lactation of approximately 344,000 MJ ME [29], as well as the feed demand for the growth of more lambs retained over winter for a flock replacement rate of approximately 25%.

During the grading up transition, the total annual sheep feed demand was constrained between approximately 55% and 65% of the total farm feed supply (Figure 5), which was achieved through varying ewe culling rates (Table 1). This contrasted with the previous use of the same model to simulate a whole flock breed transition from base Romney to second cross ¾Merino¼Romney [18] which allowed for an increase in the total annual sheep feed demand of up to 82% to reduce the time taken for transition. This reduced the size of the beef herd alongside the decrease in its feed demand. The selection of crossbred ewe lambs to enter crossbred flocks occurred at ten months of age in the transition to ¾Merino¼Romney (when wool could be tested for fibre diameter) rather than at five months in the current analysis.

### 3.4. Production

**Figure 6:**
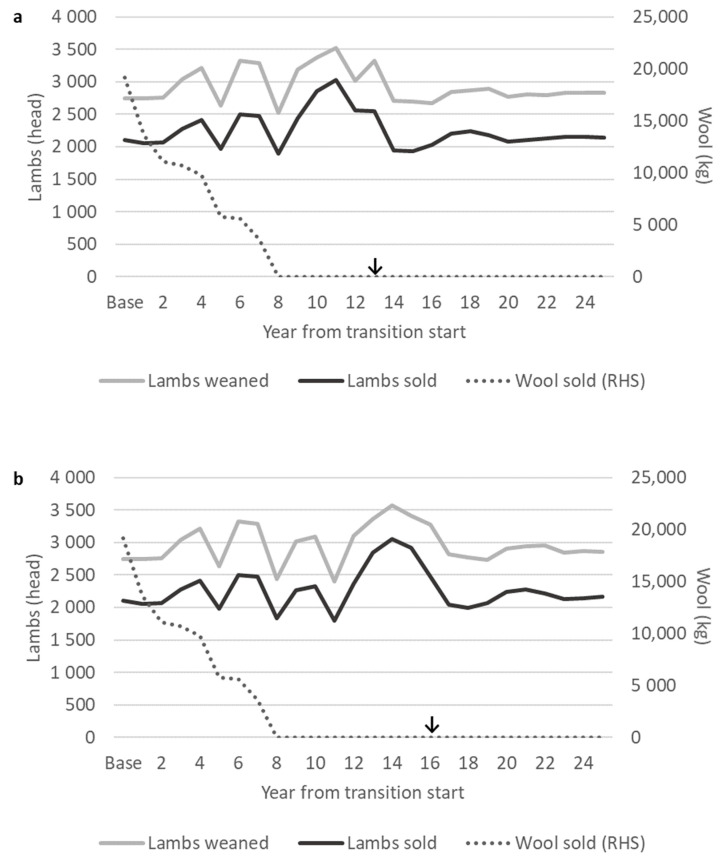
Total lambs weaned, sold, and wool sold during a grading up transition from base Romney to a desired final cross of either (**a**) ⅞W⅛R or (**b**) straightbred. RHS = right hand side vertical axis. ↓ Where the grading up transition period has finished and the desired final cross flock (⅞W⅛R or straightbred) has reached an equivalent feed demand to the base Romney flock.

Changes in numbers of weaned lambs (Figure 6) varied alongside changes in the total ewe numbers (Figure 4) due to the consistent lambing rate between crosses. The peak number of weaned lambs was more than 3500 lambs during transition scenarios. The correlation was not exact due to changes in the flock age structure, particularly the numbers of non-bred one-year-old ewes, as the relative reproductive performance of ewes varied with age (Section 2.1.1). The base Romney flock weaned 2750 lambs, and the post-transition flocks (⅞W⅛R or straightbred) weaned 81–100 more lambs (Figure 6) due to different flock size (Figure 4) and average age (Table 5). All self-replacing flocks (base Romney, post-transition ⅞W⅛R, and straightbred) had replacement rates of 25%, so the numbers of lambs sold were also slightly higher in post-transition flocks than from the base Romney flock (Figure 6). Wool was harvested only from the Romney and ½W½R flocks, so wool production decreased as the numbers of Romney and ½W½ ewes fell during the transition and was zero from eight years from transition start.

### 3.5. Economics

**Figure 7:**
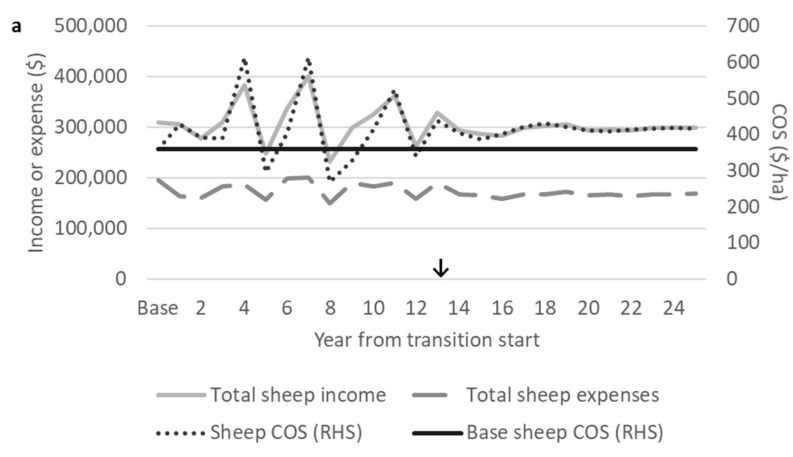

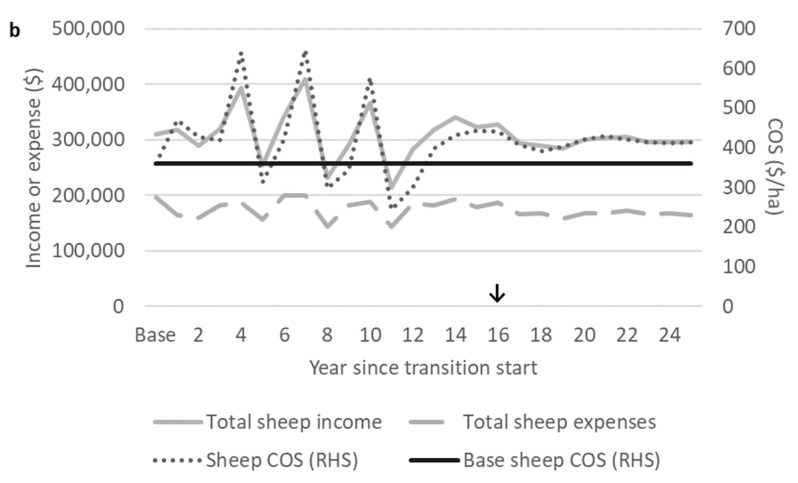
Total sheep enterprise income, expenses, and cash operating surplus (COS) during a grading up transition from base Romney to a desired final cross of either (**a**) ⅞W⅛R or (**b**) straightbred, with the base Romney sheep enterprise COS for comparison. RHS = right hand side vertical axis. ↓Where the grading up transition period has finished and the desired final cross flock (⅞W⅛R or straightbred) has reached an equivalent feeddemand to the base Romney flock.

After 25 years from transition start, when inter-year fluctuations in sheep COS flocks were relatively small, the sheep enterprise annual COS values of the self-replacing shedding ⅞W⅛R and straightbred flocks were $404 and $417/ha, respectively (Figure 7). This was a 13%–15% increase in the sheep COS for the post-transition shedding flocks compared with the base Romney flock. With an average mature ewe greasy fleece weight of 5.2 kg and a price of $2.15/kg [2], mature Romney ewes would generate $11.18 of annual income per head in wool sales. With annual shearing expenses per head of $10.98 (Table 3), the $0.20 net wool revenue per mature ewe was relatively small. Expenses for shearing by contractors for the base Romney flock comprised $38,564 in total per year. These shearing expenses did not include expenses for farmers associated with preparing sheep for shearing and maintaining shearing-related infrastructure such as the wool shed. Therefore, no longer generating income from wool sales through grading up to a fully shedding cross was not expected to have a large negative effect on the COS. There are expenses aside from shearing associated with wool harvesting, such as the maintenance of the shearing shed, which would also be eliminated through a transition to shedding flock and which were not included in this research. The ewe numbers in flocks of the desired final cross were 156–160 higher than the base Romney flock (Figure 4). With a lambing rate of 132% assumed for all crosses, the sales of lambs weaned from these additional ewes contributed to the greater COS for transition scenarios despite the associated increased expenses from more wintered ewes. The base Romney flock derived 13% of sheep enterprise income from wool sales. It should be noted that the removal of wool income for a grading up transition to shedding flock results in all sheep enterprise income being derived from sheep sales, which may increase farm financial risk from reduced income diversification.

#### 3.5.1. Cashflow During Transition

During the transition, the total ewe numbers increased to as high as 3149 from a base level of 2580 Romney ewes (Figure 4b). Changes in the total annual sheep feed demand and sheep enterprise expenses followed a similar pattern to changes in the total ewe numbers (Figure 5 and Figure 7). Sheep enterprise expenses were estimated on a per stock unit basis, so they increased with greater ewe numbers and vice versa. Sheep enterprise expenses excluding shearing were consistent for all crosses. All Wiltshire–Romney lambs were assumed not to require shearing, which may not be the case for some first cross lambs in New Zealand, for example if Wiltshire rams had a shedding score of four. The shearing of first cross lambs may incur additional expenses during the early years of transition, but this is not currently known. Anecdotal accounts of shedding flocks in New Zealand have suggested decreased labour and health expenses, such as the expenses of bringing sheep into yards for shearing and flystrike prevention. There were insufficient data to support reducing operating and animal health expenses for shedding sheep in this analysis. Future studies need to consider collecting this information so that more accurate predictions of changes in expenses can be made.

Sheep enterprise incomepeaked during transition when all remaining ewes in flocks of differing crosses were culled, reflecting the income generated through their sale (Figure 7). Changes in the sheep enterprise COS were driven by changes in income from sheep sales, and the sheep COS was mostly above the base Romney level. Sheep enterprise income was below the base Romney level of $360/ha for four years during the transition periods for the ⅞W⅛R and straightbred scenarios. This occurred when total ewe numbers were low, and the numbers of lambs weaned and sold were therefore reduced. The numbers of lambs weaned changed alongside changes in ewe numbers, as lambing rate was consistent for all crosses. The lowest sheep enterprise COS in this analysis occurred in year 11 of the transition to straightbred at $242/ha, a 33% reduction in the sheep enterprise COS and a 13% reduction in the farm COS from the base Romney level. The transition to ⅞W⅛R had a similar reduction in the sheep enterprise COS to $269/ha eight years from transition start. The lower sheep enterprise COS over several years during transition should be considered by farmers who are planning a grading up transition to self-replacing shedding flock.

#### 3.5.2. Net Present Value Analysis

Between scenarios with different desired final crosses, either ⅞W⅛R or straightbred, NPVs were very similar (within 1% of each other) for all analysed discount rates and periods (Table 6). This was not unexpected with the same sheep production (such as lambing rates and lamb weights) across all crosses, similarity in ewe numbers for the majority of the periods examined (Figure 4), and sheep feed demand constrained to be within ±5% range, followed by feed demand at the same level post-transition (Figure 5).

With a farmgate wool price of $2.15/kg, the NPVs of transition scenarios were 7% higher than the maintenance of the base Romney flock (Table 6). In the NPV analyses, earlier cashflow was most valuable. Both transition scenarios experienced a higher annual sheep COS than the base Romney flock level of $360/ha during the first four years of the transition, and the sheep COS rose as high as $611/ha (Figure 7). Though there were years during the transition where the sheep enterprise COS was below that of the base Romney flock, this occurred later, between 5 and 12 years from transition start. Additionally, the periods with a lower sheep COS occurred approximately when the proportion of farm feed consumed by sheep was also low due to low total ewe numbers; therefore, the effect of a low sheep enterprise COS on the total farm COS was buffered by the lower proportion of farm area over which the sheep enterprise COS was applied. The sheep and total farm COS were greater post-transition in the shedding flocks than the base Romney level. This also contributed to the greater NPVs of transition scenarios compared with the maintenance of the base Romney flock.

#### 3.5.3. Wool Price Sensitivity Analysis

A farmgate wool price of 2.15 $/kg greasy was used in the main analysis of this research. With a lower wool price of 1.15 $/kg, there was an increased economic benefit of grading up to a shedding flock, where NPVs of the transition scenarios averaged 12% higher than the maintenance of the base Romney flock (Table 6). With this wool price, wool production of Romney ewes generated a net loss of $5 per head from shearing expenses. Therefore, the increased economic benefit of transitioning to a shedding flock was expected. In this sensitivity analysis, it appeared the ‘break-even’ wool price was approximately 4.15 $/kg. With this wool price, the NPVs of the transition to shedding flock scenarios ranged from 99% to 100% of the NPVs of the base Romney flock (Table 6). As stated in the methods, prices for New Zealand coarse wool have decreased since the modelled 2017/18 production year and were 1.70 $/kg clean in August 2020. The farmgate wool price of 4.15 $/kg equates to a clean main fleece price of 5.70 $/kg (using fleece yield and skirting discount methods from [19]). A wool price of 5.70 $/kg clean has not occurred since 1989 [1]. Therefore, the risk of coarse wool prices rising to a level where the grading up transition would not have an economic advantage over the maintenance of the Romney flock is low. This sensitivity analysis did not include potential changes in shearing expenses, which increased in New Zealand in the second half of 2018 [52]. With greater shearing expenses, the ‘break even’ wool price in this NPV analysis would likely be higher. Conversely, if the feed demand for growth of shed wool is not negligible, the post-transition flocks would produce fewer lambs and the ‘break even’ wool price would be lower than $4.15/kg. Overall, this sensitivity analysis suggests that farmers should consider a grading up transition to a shedding flock.

#### 3.5.4. Operating Profit

**Figure 8:**
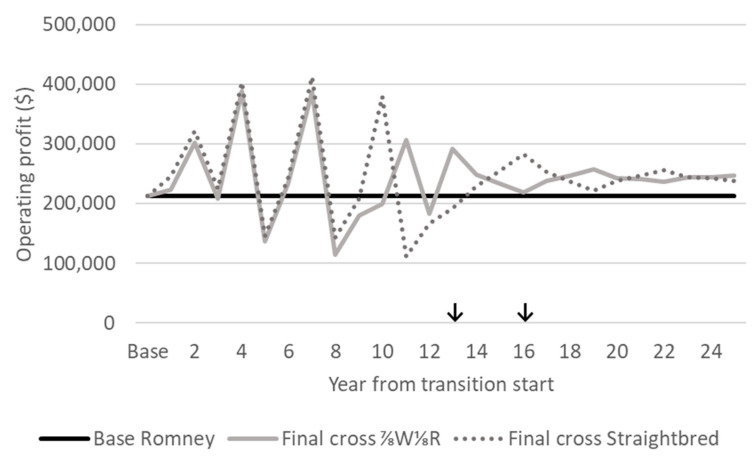
Annual operating profit during a grading up transition from base Romney to a desired final cross of either a). ⅞W⅛R or b). Straightbred, with the base Romney operating profit for comparison. ↓ Where the grading up transition period has finished and the desired final cross flock (⅞W⅛R or straightbred) has reached an equivalent feed demand to the base Romney flock.

The OPs of post transition flocks (⅞W⅛R and straightbred) were $246,742 and $236,016 for ⅞W⅛R and straightbred flocks, respectively, which were greater than that of the base Romney OP of $213,058 (Figure 8). This was due to the value of additional ewes in the larger post-transition flocks (Figure 4). OP is considered a better measure of farm financial performance than COS [46]. Therefore, the higher OP of the post-transition shedding flocks indicates a grading up transition strategy to be advantageous over the maintenance of the base Romney flock. Fluctuations in OP continued after transition was complete (i.e., 13 and 16 years from transition start) due to changes in the numbers of replacement ewe lambs and one-year-old ewes changing the total value of capital stock. When the total ewe numbers were growing (Figure 4), the value of capital stock also increased, thus increasing the OP by a larger proportion than the increase of the COS (Figure 8). Conversely, when the total ewe numbers decreased, such as when all remaining ewes of a specific cross were culled, the change in the value of capital stock decreased, which decreased the OP to a proportionally greater extent than the decrease of the COS. Therefore, peak and nadir operating profits ranged in excess of the peak and nadir COS (Figure 7) due to changes in the value of capital stock.

### 3.6. General Discussion

Production parameters excluding wool production were assumed to be consistent between purebred Romney sheep and the Wiltshire crosses due to few comparative published studies and those available being somewhat old. New Zealand Wiltshire flocks have been reported to wean heavier lambs than both Romney [11] and Perendale ewes (a dual-purpose Romney–Cheviot crossbred) [48]. New Zealand Wiltshire ewes have been reported to have similar mature liveweights, birth weights, and one-year-old liveweights to Perendale ewes, though with a slightly lower reproductive performance [48]. Therefore, overall, there were insufficient data upon which assumptions of differences in production of Romney and Wiltshire–Romney crossbred sheep on the same farm could be based. The future collection of production data for Wiltshire and Wiltshire-cross sheep in New Zealand would improve the accuracy of the model predictions.

This analysis assumed no production disadvantages of selecting crossbred ewe lambs to enter the ewe flock based on shedding score. In reality farmers may also select ewe lambs for flock replacement based on other traits such as liveweight and conformation issues. Though there are sparse data to support assumptions of changes in lamb production during the grading up transition, the performance of crossbred sheep for non-wool traits (such as lamb growth rates) may increase above the parental average due to hybrid vigour. If this were to occur, the NPVs of grading up transition scenarios would increase relative to the maintenance of the base Romney flock. These assumptions of possible changes in lamb production during and post-transition were not included in the model, and more data from Wiltshire–Romney sheep farmed in New Zealand would be helpful.

Ewes of the desired final cross were assumed to be bred with rams of a similar cross, either ⅞W⅛R rams or Wiltshire rams when the desired final cross was ⅞W⅛R or straightbred, respectively. There is potential for any production gains from hybrid vigour to be lost post-transition. However, given that no difference in non-wool traits were assumed between Romney sheep and those of the various crosses, this would not affect the current analysis. This does indicate the importance of collecting data on production in any future studies crossbreeding Wiltshire rams with Romney ewes.

An alternative strategy to reduce wool harvesting expenses for farmers of dual-purpose breed flocks in New Zealand is to shear once per year, which was not modelled in this analysis. This would usually entail farmers forgoing the winter shear and only doing a full shear in summer and crutch. With this strategy, the winter shearing expense of 4.09 $/head would be eliminated (Table 3), potentially increasing the sheep enterprise COS. Romney ewes are usually shorn twice per year, as a once-yearly shearing policy may reduce wool quality and, therefore, wool income. Additionally, not removing wool prior to spring lambing can also increase ewe death rates, decreasing lamb production with the greater casting of ewes (recumbent on their backs and unable to get up) [53]. Therefore, although the once-yearly shearing strategy was not analysed in this research, it is not necessarily a better alternative to the modelled base Romney flock scenario.

The constraining of the total annual sheep feed demand during transition to between 55% and 65% of the total farm feed from a base level of 60% would likely minimise disruptions to an on-farm beef enterprise. Increases in sheep feed demand to more than 65% of farm feed would likely necessitate sales of capital beef stock, and, if this results in a smaller breeding herd, it may limit the ability of farmers to use beef cattle for pasture management. Farmers could potentially allow for sheep feed demand to change during transition in excess of the constraints imposed in this analysis to speed up the transition period. Another factor not explored in the current analysis that could speed up the transition period is breeding ewes to first lamb at 14 months of age. Breeding ewes to first lamb a year earlier would reduce the generation interval and produce more crossbred ewes each year. A future analysis may wish to examine these potentially shorter grading up transition scenarios.

## 4. Conclusions

Results suggest that it is possible to achieve a grading up whole flock transition from purebred Romney to fully shedding majority Wiltshire based flock within 15 years without large changes in annual sheep feed demand. A crossbred ewe lamb selection intensity of 50% was sufficient to eliminate shearing expenses after seven years of the transition when utilising fully shedding Wiltshire rams. Without shearing expenses and with the absence of feed demand for wool growth, which enabled a larger flock to produce more lambs in total, the shedding ⅞W⅛R or straightbred flocks had a higher post-transition COS than the base Romney flock. The NPVs of the grading up transition scenarios were, depending on wool price, up to 12% higher than the maintenance of the base Romney flock. A sensitivity analysis suggested a ‘break even’ farmgate wool price of $4.15/kg, where NPVs of the transition scenarios and maintenance of the base Romney flock were similar. However, it is acknowledged that data on shedding sheep in New Zealand were scarce, and the collection and publication of more data would improve the accuracy of model predictions. The findings can be used to inform sheep farming industries of the potential length of time taken to finish such a grading up transition, changes in ewe numbers on-farm and cashflow during transition, and the overall potential economic benefit.

## Figures and Tables

**Table 1:** Cull rates (%) for ewes of each age class and Wiltshire–Romney cross during the whole-flock breed grading up transition from Romney to a final cross of either ⅞Wiltshire⅛Romney (⅞W⅛R) or straightbred ^1^.

	Cull Rate for Ewes of Various Age Classes (%) ^2^		
Ewe Type	Base Romney	Final Cross ⅞W⅛R	Final Cross Straightbred
Self-replacing base Romney	Two-to-five-year-olds: 19 Six-year-olds: 100	-	-
Romney	-	Two-to-four-year-olds: 4 Five-year-olds: 100	Two-to-four-year-olds: 4 Five-year-olds: 100
½W½R	-	Two-to-three-year-olds: 4 Four-year-olds: 33 Five-year-olds: 100	Two-to-three-year-olds: 4 Four-year-olds: 33 Five-year-olds: 100
¾W¼R	-	Two-to-three-year-olds: 4 Four-year-olds: 33 Five-year-olds: 100	Two-to-four-year-olds: 4 Five-year-olds: 100
⅞W⅛R	-	Two-to-five-year-olds: 4	Two-to-four-year-olds: 4 Five-year-olds: 100
Straightbred	-	-	Two-to-five-year-olds: 4
Self-replacing ⅞W⅛R	-	Two-to-five-year-olds: 19 Six-year-olds: 100	-
Self-replacing straightbred	-	-	Two-to-five-year-olds: 19 Six-year-olds: 100

^1^ Outside of the grading up transition period, self-replacing flocks (initial Romney and the final cross once the desired size was reached) had a replacement rate of 25% with death rates of 5.2%, and all six-year-old ewes were culled after weaning. For example, during the transition to a final flock of straightbred ewes, two-to-four-year-old ewes in the straightbred flock had a culling rate of 4%, and all five-year-old ewes were culled. Then once the transition period was finished, the straightbred flock was self-replacing and had a culling rate of 21% for two-to-five-year-old ewes, and all six-year-old ewes were culled. ^2^ Cull rates were applied to ewes in each specific age class before they would have moved into the next age class, e.g., when four-year-old ewes had a cull rate of 33%, 66% of live four-year-old ewes would move into the fifth ewe age class. All one-year-old ewes were assumed to not be culled.

**Table 2:** Prices for sold sheep, ewes of various age classes (Yi), and lambs sold either prime (direct to slaughter) or store (for another farmer to grow for slaughter).

Sheep Class	Timing of Sale	Value ($/head) ^1^	Price Data
*Y*_3 *to* 6_ ewes	Early December ^2^	113.73	[2]
*Y*_2_ ewes		134.64	
Prime lambs	Early February	107.44	[39]
	Mid-February	107.10	
Store lambs	Mid-February	84.00	

^1^ Prices per head for lamb sales were estimated from weekly schedule prices per kg of carcass weight and their weight at sale. ^2^ The majority of ewes were culled in early December at weaning, with a small proportion culled in June at scanning.

**Table 3:** Expenses for shearing of sheep with various shedding scores [7] based on 2017/18 New Zealand shearing contract rates reported by [41]. Romney shearing expenses consisted of per head expenses of $4.89 for a full summer shear, $4.09 for a second winter shear (with twice yearly shearing policies), and $2.00 for a full crutch ^1^.

Shedding Score	Equivalent Shearing Required	Expense ($/head)
0–0.99	Twice per year and crutch	10.98
1.00–1.99	Twice per year	8.98
2.00–2.99	Once per year	4.89
3.00–3.99	Back wool removal ^2^	2.00
4.00–5.00	Nil ^3^	0.00

^1^ Crutching is the removal of wool from the belly and breech. ^2^ Assuming only a small volume of wool requires removal from the back of the sheep, with the equivalent expense as crutching. ^3^ Assuming the volume of unshed wool is minimal and does not require removal through shearing.

**Table 4:** Predicted average shedding scores of sheep of varying crosses (e.g., ½W½R is a first cross ½Wiltshire½Romney) for a whole flock grading up transition from Romney to either ⅞Wiltshire⅛Romney or straightbred—this shows the effect of applying a selection intensity of 50% to crossbred ewe lambs.

Cross	Pre-selection Average Shedding Score with Standard Deviation	Post-selection Average Shedding Score
Romney	0 ± 0	0
½W½R	2.5 ± 0.95	3.14
¾W¼R	4.07 ± 0.33	4.29
⅞W⅛R	4.64 ± 0.23	4.8
Straightbred	4.9 ± 0.10	4.97

**Table 5:** Average age of ewes in flock of different crosses at various time points (T) from the start of a grading up transition from Romney to a desired final cross of either ⅞W⅛R or straightbred.

	Final Cross ⅞W⅛R		Final Cross Straightbred	
Cross	T (years)	Flock Average Age (years)	T (years)	Flock Average Age (years)
Romney	T0	3.45	T0	3.45
	T4	4.46	T4	4.46
½W½R	T7	4.02	T7	4.02
¾W¼R	T11	4.48	T9	3.77
⅞W⅛R	T13	3.92	T15	4.99
	T25	3.48	-	-
Straightbred			T16	3.45
			T25	3.40

**Table 6:** Net present values of annual farm cash operating surplus (sheep and beef enterprises combined) of the maintenance of the status quo Romney flock compared with the grading up transition from Romney to a desired final cross of either ⅞W⅛R or straightbred. Net present analyses were also conducted for each scenario with varying farmgate wool prices.

Scenario	15 Years ^1^		25 Years	
	6% Discount Rate ^2^	10% Discount Rate	6% Discount Rate	10% Discount Rate
**Wool Price $2.15/kg Greasy ^3^**				
Base Romney	3,622,240	2,910,060	4,660,704	3,407,450
Final cross ⅞W⅛R	3,875,321	3,115,634	4,980,752	3,645,054
Final cross straightbred	3,871,501	3,116,354	4,978,018	3,646,396
**Wool price $1.15/kg Greasy**				
Base Romney	3,378,108	2,713,927	4,346,581	3,177,795
Final cross ⅞W⅛R	3,772,669	3,021,288	4,878,100	3,550,708
Final cross straightbred	3,806,774	3,046,451	4,910,665	3,575,082
**Wool Price $3.15/kg Greasy**				
Base Romney	3,872,050	3,110,753	4,982,132	3,642,447
Final cross ⅞W⅛R	3,978,503	3,210,510	5,083,974	3,739,946
Final cross straightbred	4,012,361	3,235,480	5,116,252	3,764,111
**Wool Price 4.15 $/kg Greasy**				
Base Romney	4,116,182	3,306,886	5,296,255	3,872,103
Final cross ⅞W⅛R	4,092,195	3,311,438	5,223,204	3,853,103
Final cross straightbred	4,097,288	3,319,807	5,218,817	3,857,043

^1^ NPV analysis was conducted for either 15 (focusing only on the grading up transition period) or 25 years (including the time taken for ewe numbers in each age class of the final cross flock to stabilise). ^2^ Discount rates representing current 2018 interest rates of 6% [44] and long-term business lending interest rates of 10% [43]. ^3^ Wool price used in the main part of the analysis for the 2017/18 production year [2].

## Supplementary Materials

The following are available online at https://www.mdpi.com/2076-2615/10/11/2066/s1. The supplementary material provides more detail on estimations of sheep feed demand.
