# Supplementary material for: Modelling a Transition from Purebred Romney to Fully Shedding Wiltshire–Romney Crossbred
Source: Animals (Basel). 2020 Nov 7;10(11):2066. doi: 10.3390/ani10112066 (PMC7695207; doi:10.3390/ani10112066)
Supplement: Supplementary file 1 [file animals-10-02066-s001.pdf]

Supplementary: The material in this supplementary file provides further detail on the equations and parameters used to estimate feed demand (in megajoules of metabolisable energy) in the study.

Sheep feed demand for daily maintenance energy (ME_m) was calculated from Equation S1 [1].

$$ME_m = \left[0.28 \times \frac{LW^{0.75} \times e^{-0.03 \times i}}{0.02 \times Q + 0.5}\right] \times 1.1 \quad [S]$$

Where LW = liveweight (kg) and Q = pasture quality measured as MJ ME/kg DM, assumed to be 10 MJ ME/kg DM which is considered a medium quality of pasture on New Zealand sheep and beef farms [2]. Mature ewe (two to six year old) average liveweight was 65 kg, losing 2 kg in spring during lactation which was regained prior to autumn breeding. Liveweight of replacement ewes was assumed to average 75% of mature ewe liveweight when entering Y_1 at twelve months of age [3, 4]. Liveweight values used to calculate maintenance demand for sheep younger than Y_2 were averages for that class of animal. For example, single-born prime lambs were weaned at 30 kg (Section 2.3 in manuscript) and sold for slaughter at 44 kg liveweight, hence demand for maintenance between weaning and slaughter was based on an average liveweight of 37 kg. Feed demand for liveweight gain was 55 MJ ME required for each kg of liveweight gain, and 35 MJ ME converted from each kg of liveweight loss [5].

Feed demand for gestation (ME_G) and lactation (ME_L) were calculated per lamb according to Equations S2 and S3 [5]. The average New Zealand lamb loss rate (from scanning to weaning) of 16% [6, 7] was used alongside the lambing rate of 132% [8] to estimate numbers of lamb foetuses for gestation energy demand calculations.

$$ME_G = 49 \times b + 7 \quad [S2]$$

And $$ME_L = N \times [51.4 \times L + 134.7 \times \alpha - 1808] \quad [S3]$$

Where b is lamb birthweight (kg; values in Section 2.3 of manuscript), N is the adjustment parameter for birth rank (N = 1 for single-born lambs and N = 1.35 for multiples; [1]), L = lamb liveweight at weaning (kg; values in Section 2.3 of manuscript), and α = lamb age at weaning in weeks.

Average ewe greasy fleece weight (kg; W in section 2.2 in manuscript) was used to calculate flock daily wool growth (G) in g/sheep/day. Feed demand for wool growth (ME_w) was estimated using the wool growth Equation S4 [1].

$$ME_w = 0.13 \times (G - 6) \quad [S4]$$

1. CSIRO. *Nutrient Requirements of Domesticated Ruminants*; CSIRO Publishing: Australia, 2007.
2. Waghorn, G.C.; Burke, J.L.; Kolver, E.S. Principles of feeding value. In *Pasture and Supplements for Grazing Animals.* Rattray, P.V., Brookes, I.M., Nicol, A.M., Eds.; New Zealand Society of Animal Production Occasional Publication: city, New Zealand, 2007, pp. 151–172.

3. Thomson, B.; Muir, P.; Smith, N. Litter size, lamb survival, birth and twelve week weight in lambs born to cross-bred ewes. Proceedings of the New Zealand Grassland Association 66, Ashburton, New Zealand, day month 2004, 233-237.
4. Corner, R.; Mulvaney, F.; Morris, S.; West, D.; Morel, P.; Kenyon, P. A comparison of the reproductive performance of ewe lambs and mature ewes. *Small Rumin. Res.* **2013**, 114, 126-133. doi.org/10.1016/j.smallrumres.2013.05.018
5. Nicol, A.M.; Brookes, I.M. 2007. The metabolisable energy requirements of grazing livestock. In *Pasture and Supplements for Grazing Animals.* Rattray, P.V., Brookes, I.M., Nicol, A.M., Eds.; New Zealand Society of Animal Production Occasional Publication: city, New Zealand, day month 2007, pp. 151–172.
6. Dalton, D.; Knight, T.; Johnson, D. Lamb survival in sheep breeds on New Zealand hill country. *N.Z. J. Agric. Res.* **1980**, *23*, 167-173. doi.org/10.1080/00288233.1980.10430783
7. Amer, P.; McEwan, J.; Dodds, K.; Davis, G. Economic values for ewe prolificacy and lamb survival in New Zealand sheep. *Livest. Prod. Sci.* **1999**, 58, 75-90. doi.org/10.1016/S0301-6226(98)00192-4.
8. Benchmark your farm. Available online: https://beeflambnz.com/data-tools/benchmark-your-farm. (accessed on 05/02/2020).
